# Delays in Cardiovascular Emergency Responses in Africa: Health System Failures or Cultural Challenges?

**DOI:** 10.9745/GHSP-D-24-00092

**Published:** 2024-10-29

**Authors:** Kofi Tekyi Asamoah, Alfred Doku, Florence Akumiah, Eugene Ampofo, Fiifi Duodu, Francis Agyekum, Mohammed Hafez, Joseph Akamah, Nicholas Ossei-Gerning, James Baligeh Walter Russell, Charles Agyemang

**Affiliations:** a National Cardiothoracic Centre, Korle-Bu Teaching Hospital, Accra, Ghana.; b Department of Medicine and Therapeutics, Korle-Bu Teaching Hospital, Accra, Ghana.; c Department of Pediatrics, Yale School of Medicine, New Haven, CT, USA.; d Department of Public and Occupational Health, University of Amsterdam Medical Centre, University of Amsterdam, Netherlands.; e University of Ghana Medical School, Accra, Ghana.; f National Heart Institute, Giza, Egypt.; g International Maritime Hospital, Tema, Ghana.; h Nashville General Hospital, Meharry Medical College, Nashville, TN, USA.; i The Grange University Hospital, South Wales, United Kingdom.; j Faculty of Clinical Sciences and Dentistry, College of Medicine and Allied Health Sciences, University of Sierra Leone, Freetown, Sierra Leone.; k Division of Endocrinology, Diabetes, and Metabolism, Department of Medicine, Johns Hopkins University School of Medicine, Baltimore, MD, USA.

## Abstract

Delays in receiving care for debilitating cardiovascular emergencies, such as myocardial infarction and stroke, are multifaceted and include personal, systemic, and health facility-related factors, which must all be addressed to successfully improve cardiovascular emergency outcomes.

## Key Messages

- Cardiovascular emergencies contribute significantly to morbidity and mortality in Africa, partly perhaps due to a combination of time-related delays in appreciating symptoms, getting to an intervention-capable facility, and prevailing emergency care systems.
- Drawing experiences from across Africa, we discuss unique limitations to prompt and appropriate management of cardiovascular emergencies in the African setting.
- Investing in multisectoral collaboration is required to improve the status quo of cardiovascular emergency care.
- Community education is important in ensuring that patients who suffer cardiovascular emergencies recognize their symptoms and take early steps to seek prompt, appropriate medical care.
- Well-defined emergency systems are required to facilitate the transport of patients with cardiovascular emergencies to appropriate hospitals. This involves improving road networks, training emergency responders, and having ambulances equipped with electrocardiograms and thrombolytic drugs, among others. These services should also be able to directly activate hospital emergency services before arrival, ensuring a seamless continuity of care.
- Health care providers on duty should be abreast with recognizing cardiovascular emergencies and the local avenues for optimal care, minimizing hospital-related delays in such patients.

## INTRODUCTION

Noncommunicable diseases are the leading cause of morbidity and mortality in low- and middle-income countries (LMICs).^1^ Among noncommunicable diseases, the incidence of cardiovascular disease (CVD) has increased by about 50% over the last 30 years in sub-Saharan Africa (SSA).^2^ The incidence and outcomes of CVD in LMICs are influenced by factors, including urbanization and related lifestyle changes, belief systems and practices, level of education, and economic and political systems, translating into poor health-seeking behavior and emergency health care systems.^1^^,^^2^ The scope of cardiovascular emergencies includes hypertensive emergencies, acute heart failure (HF), acute coronary syndromes (ACS), stroke, aortic dissection, pulmonary embolism, cardiac tamponade, and cardiac arrest. In 2017, ischemic heart disease accounted for about 5% of all deaths and 40% of cardiovascular deaths in SSA, commonly presenting as HF.^2^ HF has an estimated 6-month mortality of 18%, a 5-year survival rate of 50%, and a 30-day rehospitalization rate of 25% in SSA.^2^ The surge in cardiovascular emergencies and their sequelae has been driven by increases in the incidence of cardiovascular risk factors, like hypertension, diabetes, and adoption of a Western lifestyle.^2^^,^^3^ The pooled prevalence of hypertension in SSA, according to the Pan-African Society of Cardiology, is about 30%, with figures from individual studies ranging from 19.3% to 39.6%.^4^ The role of hypertension in atherosclerotic CVDs, such as ACS and stroke, has been demonstrated in the multinational INTERHEART and INTERSTROKE studies.^5^

Timely intervention is critical in ACS and stroke to prevent complications. Immediate action is necessary to achieve maximum benefit within a narrow window of opportunity. Prompt identification, diagnosis, and appropriate therapeutic measures are crucial for successful management. Delay in intervention may result in significant morbidity and mortality, underscoring the importance of early and decisive action in these clinical scenarios.^6^^,^^7^ The current standard of care for patients with ACS is to have revascularization therapy, preferably within 12 hours of symptom onset and within 120 minutes of diagnosis by a medical professional, with a door-to-needle time not exceeding 30 minutes from first contact with the health facility.^6^ However, for infarctive strokes, revascularization therapy is recommended within a maximum of 4.5 hours of symptom onset, with earlier times being preferred.^7^ For ACS, the inability to reach a facility capable of performing a percutaneous coronary intervention (PCI) within 120 minutes is an indication to offer fibrinolytic therapy, preferably within 10 minutes of taking the decision.^6^ Failing to adhere to these timelines puts the patient at risk of complications, such as heart failure, malignant cardiac arrhythmias, permanent neurological deficits, and, ultimately, cardiac arrest. Therefore, it is essential to ensure prompt and appropriate action is taken to mitigate these risks.

Cardiac arrest is the ultimate cardiovascular emergency, denoting a sudden cessation of mechanical activity of the heart, with or without the presence of electrical activity. These patients have no active circulation, and failure to promptly restore circulation results in ischemia to major organs, culminating in multi-organ failure and death. Irreversible anoxic brain injury begins after 4 minutes of cerebral ischemia, underscoring the importance of time in resuscitation efforts. ACS is a common cause of cardiac arrest, often due to related cardiac arrhythmias.^6^ Other common causes are collectively referred to as the “Hs” (hypovolemia, hyperkalemia, hypokalemia, hypoxia, acidosis, and hypothermia) and “Ts” (tension pneumothorax, cardiac tamponade, coronary and pulmonary thrombosis, and toxins).^8^ Management of cardiac arrest involves early defibrillation based on the underlying heart rhythm, fast and rhythmic chest compressions to maintain perfusion, rescue breaths to maintain oxygenation, and the use of emergency medications, such as adrenaline and amiodarone, as appropriate.^8^

In clinical practice, there have been several situations in LMICs, including SSA, where sufferers of ACS and infarctive stroke report to the intervention-capable facilities outside the ideal timelines, precluding the administration of standard therapy, which results in greater expenditure on treatment and rehabilitation.^9^^–^^11^ The reasons for these delays in arrival are multifactorial and broadly categorized into patient-related and health system delays.

Considering the aforementioned, it is prudent to question whether personal and systemic deficiencies weigh equally in delayed optimal therapy for cardiovascular emergencies in Africa. Given the ravaging effects of these emergencies on families, communities, and the economy, what are the most appropriate measures to mitigate them?

What are the most appropriate measures to mitigate personal and systemic deficiencies in receiving optimal care for cardiovascular emergencies?

## CASE VIGNETTES IN AFRICA

### Case 1

A middle-aged man with hypertension and diabetes was noticed to have a sudden onset of right-sided upper and lower limb weakness. He was sent to the nearby health facility about 4 hours after onset of symptoms, where he had a head computed tomography (CT) scan done within an hour, showing an infarctive stroke. He was detained and given soluble aspirin 300 mg and rosuvastatin 40 mg immediately and started on nifedipine 40 mg daily. Eight hours later, he arrived at a tertiary facility for further management following a referral from the initial facility he visited.

### Case 2

An elderly woman with hypertension and diabetes for 2 years, with poor treatment adherence, began to experience sudden-onset chest pain at night (1:00 am), for which she took an antacid on suspicion of heartburn. After 3 hours of persistent pain, she went through 2 facilities before being referred to a tertiary facility and arrived there 14 hours after onset of chest pain. Her first electrocardiogram (ECG) was done at the tertiary facility and showed an inferolateral ST-segment elevation myocardial infarction. The total time taken to see the cardiology team was 28 hours after the onset of pain due to routine hospital processes. The woman was offered primary revascularization therapy with PCI, as she had developed acute left ventricular failure with ongoing chest pain, but she declined due to an inability to afford the equivalent of US$3500 cost. Therefore, she was managed conservatively with medical therapy.

## CAUSES AND CLINICAL RELEVANCE OF TIME DELAYS IN MANAGING CARDIOVASCULAR EMERGENCIES

CVD management guideline recommendations for physicians recommend intervention within specific time frames to maximize outcomes.^6^^,^^7^ Unfortunately, both patients in the described cases reported to the hospital many hours after onset of symptoms, limiting their therapeutic options. The first patient arrived outside the time limit for considering thrombolytic therapy for infarctive stroke.^7^ Late arrival at the hospital has been described by various authors across Africa: in Senegal,^12^ Morocco,^13^ South Africa,^14^ and Egypt.^15^ Pre-hospital delays have been attributed to various factors, including poor understanding of symptoms by patients,^13^^,^^16^ being married, female gender,^12^ the absence of emergency services to transport patients from home on good road networks to the hospital, and absence of a centralized referral system.^12^^,^^13^ Similar observations have been made among patients with ACS. The second patient interpreted her chest pain as heartburn, which is a common misinterpretation of chest pain as stated in a study by Stassen et al.^16^ Just as this patient presented outside the recommended 12-hour window,^6^ various studies have shown this to be the trend in the African setting as demonstrated in Egypt,^10^ Burkina Faso,^17^ and Senegal.^18^ Her presentation with acute HF was, therefore, unsurprising.

Though the patient in case 1 got a diagnostic CT scan of the brain done, delays in this process were demonstrated. Similarly, there have been delays in getting ECGs to aid physicians in diagnosing and instituting management for patients reporting with ACS. This may either be due to unavailable diagnostic logistics in the primary facilities or health workers failing to attach the level of urgency that befits the situation. The absence of a clear plan for such emergencies results in some health workers not knowing what roles to play, how urgently to do so, and which channels to activate for prompt care.

While the availability of reliable emergency services is low in SSA, there is varied patronage of these services even when present, from as low as about 4% in Senegal^12^ to 31% in South Africa.^14^ Emergency services are provided by trained paramedics who, in some jurisdictions, can identify emergencies and offer life-saving therapy en route to the appropriate facility. These paramedics can perform ECGs, administer thrombolytics where appropriate,^16^ and liaise with PCI services in hospitals to ensure that relevant interventional teams are on standby awaiting the patient’s arrival. This is absent in SSA and is described as a fragmented coronary care network.^16^ As a result, patients may arrive in hospitals without any prior arrangements made, prolonging the time to receive the required care. Non-emergency vehicles, which are the more frequently used type of transportation, do not have these privileges, while the absence of sirens on these vehicles makes them subject to the prevailing vehicular traffic conditions. Poor roads in rural and semi-urban areas in most African countries also prolong the time taken to arrive at facilities capable of these services. This contributes to delays in arriving at the relevant health facilities to obtain the required help.

On arrival in the hospital, triage formalities delay revascularization therapy as reported in South Africa,^16^ where health workers confirmed that patients with chest pain are triaged and seen as usual. Brain and heart tissue lost cannot be regenerated, requiring that these ACS and strokes be prioritized above the regular bureaucratic systems to prevent life-threatening complications. ECGs and head CT scans (depending on which is appropriate for the suspected diagnosis) for such patients must be done as part of the initial assessment for prompt therapy where required.

A unique source of delay is observed in the second case: inability to afford therapy. The cost of these interventions is high in SSA.^18^ They are priced in U.S. dollars, a currency against which most African currencies perform poorly. In Ghana, a coronary angiogram with a stent costs a minimum of US$3500, with thrombolytic therapy costing about 30% of this. Considering a minimum wage of less than US$2 per day, it can be appreciated how steep the costs of these services are. The second patient was denied revascularization therapy as she could not afford PCI, which exposed her to the full extent of morbidity associated with myocardial infarction.^6^

## IMPROVING THE STATUS QUO

We propose that to effectively tackle the delays related to cardiovascular emergencies, they should be addressed in “3 delay domains”: personal/patient-related delays, systemic delays (which encompasses provisions in place to ensure that a person who decides to report to the hospital has a safe means of transportation to arrive at the appropriate facility with minimum delay), and health facility-related delays.

### Improving Patient-Related Delays

The foremost step in improving the emergency response is to improve the health-seeking habits of individuals, as people cannot be helped if they do not appreciate the problem and take steps to seek help. The capacity of the population to recognize CVDs can be built by empowering the individual through education on the risk factors, signs, and symptoms. This will help give individuals a sense of urgency about their symptoms and make them seek prompt care. Collaborations among various health care worker cadres, such as doctors, nurses, public health practitioners, and community health workers, are needed to realize this.

The foremost step in improving the emergency response is to improve individuals’ health-seeking habits.

First, the role of community engagement cannot be overemphasized. Many African countries have a strong sense of family and community. They particularly submit to the counsel of religious and traditional cultural leaders. Therefore, it is sound that a community-based approach be prioritized to reach the masses through educative efforts. Identifying community champions to reinforce educative efforts is beneficial, as shown by Chandraratne et al.^19^ in Sri Lanka. Trained health care workers can also visit churches, mosques, and other places of worship to educate worshippers on cardiovascular emergencies. Culture and religion are vital in shaping habits in SSA, and including traditional authorities and religious leaders in educational efforts will help address delays. Furthermore, this will help improve certain cultural practices that may potentially be harmful to the health of community members. In certain African communities, for example, religious and cultural beliefs require some women to seek approval from their husbands before going to the hospital.^20^^,^^21^ Therefore, the husbands’ perception of time plays a role in determining how quickly they arrive at the hospital. Religious and community leaders can help address this cause of African time delay.

To maximize coverage, the media (print and broadcast media) also has a key role to play in educational efforts. This will require planning to ensure appropriate information is being circulated and suffices as a medium-long term approach. Media education is frequently limited to specific landmark events, such as World Hypertension Day and World Heart Day. This is inadequate in attempting to improve health behavior for cardiovascular emergencies. Beyond this, media outlets can circulate verified information to patrons, ensuring a more consistent flow of education across the country. Relevant specialists can craft short messages/reminders that can be broadcast intermittently as is done with other advertisements. Furthermore, there should be occasional programs (physical and/or virtual) with question-and-answer segments where resource persons can educate and clarify/debunk myths related to emergency cardiovascular care.

### Improving Systemic Causes of Delay

The first step in improving systemic delays is to constitute an emergency care network,^16^ where emergency responders know which facilities to move to and can contact them directly, knowing that the receiving facility will mobilize a response team to stand by for their arrival. First responders must be trained to understand the urgency of such cases, as this is key to survival. This must be undertaken using an intentionally designed curriculum to ensure uniformity and reproducibility over the years.

After improving the capacity of first responders, they need to be equipped to perform relevant tests, like ECGs, where necessary when responding to emergency calls. Whether ACS or stroke, the trained paramedics will be able to determine the appropriate level of facility to transport the patient to without undue delay, improving outcomes. Lists of facilities with the capacity to manage cardiovascular emergencies must be prepared and updated regularly as their capacity changes. This is particularly important as Damon et al.^12^ realized that most patients are first sent to facilities incapable of offering optimal care, and this contributes substantially to the delays observed for health emergencies in general. Stassen et al.^16^ highlighted the importance of emergency services sending patients to PCI-capable facilities rather than the nearest hospital, as this practice does not improve outcomes.

There must also be improvements in the road networks and dedicated emergency phone lines that offer direct access to patients who require these services. Furthermore, this will require increasing capacity so that the responsibility does not fall on the same select few with each emergency.

### Improving Hospital-Based Factors

Health workers in both public and private sectors also require training in recognizing and appropriate handling of cardiovascular emergencies. They need to appreciate that when such patients arrive, premium should not be placed on making financial deposits and payments before they are seen. They must triage with haste, perform an ECG, or request for a head CT scan as appropriate and call the relevant team directly rather than adding to the regular pool of emergencies to be seen eventually. Where interventions are not available in their facilities, they must appreciate the need for urgent referral in liaison with the intervention-capable facility. Failing to appreciate such time-sensitivity can lead to loss of lives.

There must be more interventional cardiologists, neurologists, and other specialized doctors who form the core of these cardiovascular emergency response teams. As of 2018, there were about 2,000 cardiologists distributed unequally across SSA.^22^ This number includes all general and specialized cardiologists, with the former being the larger number. There were also about 22 cardiothoracic centers, with little expertise to provide the required services.^22^ There are also few neurologists, with a ratio of 0.03 per 100,000 population compared to Europe’s 8.45 per 100,000.^23^ This is inadequate to meet the cardiovascular needs of the continent and contributes to poor outcomes and recovery. Therefore, there is a heavy reliance on specialists visiting from higher-income countries,^2^ which is unsustainable. Correcting this requires investment in training to improve the skills of staff at all levels.

### Government Commitment

Substantial investments from governments and the private sector are required to implement solutions to surmount issues pertaining to both personal delays and inefficient emergency response networks. First, governments need to invest in mass educational efforts and training first responders to be abreast with current standards and care practices. Settlements must be planned with good vehicular access for easy location in emergencies. There must then be ample investment in comprehensive insurance packages that allow citizens of countries in SSA to access these services without the need for financial deposits to increase access to care through affordability. Cost subsidization is achievable, as the Ugandan government shares 50% of the cost of procedures such as these with citizens,^24^ while Sudanese enjoy free PCI and thrombolytic care to patients who meet standard criteria in the management guidelines.^25^

Substantial investments from governments and the private sector are required to implement solutions to surmount issues pertaining to both personal delays and inefficient emergency response networks.

## CONCLUSION AND RECOMMENDATIONS

Cultural characteristics, which influence personal health behavior, are key causes of delays in accessing emergency cardiovascular services. However, it is evident that systemic issues synergize with these factors in limiting prompt reportage to hospitals and start of treatments.

Improving the standard of care requires multidisciplinary collaboration, including educating the populace on symptoms of cardiovascular emergencies, like ACS and stroke, and what to do during an event. Improving public awareness and referral systems is crucial to reduce prehospital fatalities. An improved understanding of cardiovascular emergencies and their attendant health implications will reduce delays. There must also be investment in the emergency services to improve their reliability and professionalism and bolster their role in the emergency cardiovascular care network. However, this cannot be achieved without ample investment from governments and the private sector, as the costs are too high for the average citizens to afford in SSA.

## Notes

**Peer Reviewed**

**First Published Online:** October 17, 2024.
